# Phytochemicals: Extraction, Isolation, and Identification of Bioactive Compounds from Plant Extracts

**DOI:** 10.3390/plants6040042

**Published:** 2017-09-22

**Authors:** Ammar Altemimi, Naoufal Lakhssassi, Azam Baharlouei, Dennis G. Watson, David A. Lightfoot

**Affiliations:** 1 Department of Food Science, College of Agriculture, University of Al-Basrah, Basrah 61004, Iraq; 2 Department of Plant, Soil and Agricultural Systems, Plant Biotechnology and Genome Core-Facility, Southern Illinois University at Carbondale, Carbondale, IL 62901, USA; naoufal.lakhssassi@siu.edu (N.L.); baharlouei@siu.edu (A.B.); dwatson@siu.edu (D.G.W.); ga4082@siu.edu (D.A.L.)

**Keywords:** antimicrobial, antioxidants, medicinal plants, BHT

## Abstract

There are concerns about using synthetic phenolic antioxidants such as butylated hydroxytoluene (BHT) and butylated hydroxyanisole (BHA) as food additives because of the reported negative effects on human health. Thus, a replacement of these synthetics by antioxidant extractions from various foods has been proposed. More than 8000 different phenolic compounds have been characterized; fruits and vegetables are the prime sources of natural antioxidants. In order to extract, measure, and identify bioactive compounds from a wide variety of fruits and vegetables, researchers use multiple techniques and methods. This review includes a brief description of a wide range of different assays. The antioxidant, antimicrobial, and anticancer properties of phenolic natural products from fruits and vegetables are also discussed.

## 1. Introduction

Many antioxidant compounds can be found in fruits and vegetables including phenolics, carotenoids, anthocyanins, and tocopherols [1]. Approximately 20% of known plants have been used in pharmaceutical studies, impacting the healthcare system in positive ways such as treating cancer and harmful diseases [2]. Plants are able to produce a large number of diverse bioactive compounds. High concentrations of phytochemicals, which may protect against free radical damage, accumulate in fruits and vegetables [3]. Plants containing beneficial phytochemicals may supplement the needs of the human body by acting as natural antioxidants [4]. Various studies have shown that many plants are rich source of antioxidants. For instance, vitamins A, C, E, and phenolic compounds such as flavonoids, tannins, and lignins, found in plants, all act as antioxidants [3]. The consumption of fruits and vegetables has been linked with several health benefits, a result of medicinal properties and high nutritional value [5]. Antioxidants control and reduce the oxidative damage in foods by delaying or inhibiting oxidation caused by reactive oxygen species (ROS), ultimately increasing the shelf-life and quality of these foods [6]. Beta carotene, ascorbic acid, and many phenolics play dynamic roles in delaying aging, reducing inflammation, and preventing certain cancers [7]. Increasing the consumption of fruits and vegetables has been recommended by many agencies and health care systems throughout the world [8].

The objective of this paper is to provide a review of phytochemical studies that have addressed extracting, measuring and identifying bioactive compounds of plants. This review includes an overview of the lipid oxidation process, details of plants known to be antioxidant and antimicrobial sources, phenolic compounds, antioxidants from vegetables and fruits, cancer prevention, extraction techniques for phenolic compounds, isolation and purification of bioactive molecules, and techniques for structural classification of bioactive molecules. 

## 2. Methods Used for Bioactive Compound Extraction, Isolation, and Purification 

### 2.1. Extraction of Phenolic Compounds Using Solvents

Scientists have studied and analyzed the impact of different types of solvents, such as methanol, hexane, and ethyl alcohol, for the purpose of antioxidant extraction from various plants parts, such as leaves and seeds. In order to extract different phenolic compounds from plants with a high degree of accuracy, various solvents of differing polarities must be used [9]. Moreover, scientists have discovered that highly polar solvents, such as methanol, have a high effectiveness as antioxidants.

Anokwuru et al. reported that acetone and *N*,*N* dimethylformamide (DMF) are highly effective at extracting antioxidants, while Koffi et al. found that methanol was more effective in at a large amount of phenolic contents from walnut fruits when compared to ethanol [10,11,12].

It has been reported that ethanolic extracts of Ivorian plants extracted higher concentrations/amount of phenolics compared to acetone, water, and methanol [11]. Multiple solvents have been commonly used to extract phytochemicals, and scientists usually employed a dried powder of plants to extract bioactive compounds and eliminate the interference of water at the same time.

Solvents used for the extraction of biomolecules from plants are chosen based on the polarity of the solute of interest. A solvent of similar polarity to the solute will properly dissolve the solute. Multiple solvents can be used sequentially in order to limit the amount of analogous compounds in the desired yield. The polarity, from least polar to most polar, of a few common solvents is as follows: Hexane < Chloroform < Ethylacetate < Acetone < Methanol < Water.

### 2.2. Microwave-Assisted Extraction (MAE)

MAE has attracted the attention of researchers as a technique to extract bioactive compounds from a wide variety of plants and natural residues [12]. Microwaves have electromagnectic radiation that occurs at frequencies between 300 MHz to 300 GHz, and wavelengths between 1 cm and 1 m. These electromagnetic waves consist of both an electrical field and a magnetic field. These are described as two perpendicular fields. The first application of microwaves was to heat up objects that can absorb a part of the electromagnetic energy and convert it into heat. Commercial microwave instruments commonly use the frequency 2450 MHz, which corresponds to an energy output of 600–700 Watts [13].

Recently, advanced techniques have become available to reduce the loss of bioactive compound without increasing the extraction time. Therefore, microwave-assisted extraction is demonstrated to be a good technique in multiple fields, especially in the medicinal plant area. Moreover, this technique reduced the losses of the biochemical compounds being extracted [14]. Microwave-assisted extraction (MAE) has been used as an alternative to conventional techniques for the extraction of antioxidants because of its ability to reduce both time and extraction solvent volume [15]. In fact, the main objective of using MAE is to heat the solvent and extract antioxidants from plants with a lesser amount of these solvents [13].

Li et al. reported that conventional methods using various solvents presented less antioxidant activity and phenolic content than MAE [16]. Therefore, the finding confirmed that MAE was more effective at increasing antioxidant activity by measuring ferric reducing antioxidant power (FRAP), oxygen radical absorbance capacity (ORAC), and total phenolic content (TPC). The efficiency of the microwave extraction can be changed through some factors such as extraction temperature, solvent composition, and extraction time. The extraction temperature was usually studied more than other factors due to its ability to increase the efficiency of the microwave extraction. Tsubaki et al. reported that 170 °C was the most effective temperature for extracting phenolic compounds from Chinese tea. In addition, increasing the extraction temperature beyond this point resulted in a reduced extraction yield [17]. Recently, Christophoridou et al. used a new microwave-assisted extraction (MAE) process, which converts energy to heat, thereby cooperating with solvents in order to extract a specific compound [18]. Williams et al. showed many advantages of MAE, including lower solvent consumption, shorter extraction times, and higher sensitivity towards target molecules [19]. A comparison of some antioxidant methods used has been provided in Table 1.

### 2.3. Ultrasonic-Assisted Extraction

Ultrasound-assisted extraction (UAE) has been used in diverse applications of food-processing technology to extract bioactive compounds from plant materials [19]. Ultrasound, with levels greater than 20 kHz, is used to disrupt plant cell walls, which helps improve the solvent’s ability to penetrate the cells and obtain a higher extraction yield. UAE can use a low operating temperature through processing, maintaining a high extract quality for compounds. UAE is known to be one of the easiest extraction techniques because it uses common laboratory equipment such as an ultrasonic bath. In this technique, a smashed sample is mixed with the suitable solvent and placed into the ultrasonic bath, while temperature and extraction time are controlled [20].

UAE of various organic and inorganic samples can use a wide range of solvents. Common equipment used in ultrasound-assisted extraction includes an ultrasonic bath and an ultrasonic probe system. Unfortunately, ultrasonic probe has two main negative properties mainly related to experimental repeatability and reproducibility [21].

Tabaraki et al. noted that green technology is necessary to protect the environment from toxic substances [22]. Therefore, extraction of phenolic compounds by ultrasound has grown during recent years due to its role in reducing the amount of solvent and energy used. Corrales et al. have shown that UAE can break down plant tissue and work properly during the production process and release of active compounds in solvents with a high efficiency [21]. Results showed an increase in antioxidant activity from 187.13 μmol TE g^−1^DM to 308 μmol TE g^−1^DM by using UAE as an effective method to extract antioxidants from different sources. Recently, Albu et al. studied and applied the use of ultrasound to extract phenolic compounds from rosemary [23]. Multiple criteria have been compared including ultrasonic bath extractions, ultrasonic probe system, a shaking water bath at various temperatures, and different solvents to select the most efficient method. In all situations, the operation time was dramatically decreased by applying and using the ultrasonic bath and probe systems.

Similar behavior was reported by Cho et al. when extracting resveratrol from grapes [24]. In another study, Barbero et al. suggested the use of ultrasound in different industries because of its positive effects in the extraction of capsaicinoids of hot peppers [25]. Moreover, the ultrasonic method had the ability to decrease the degradation of phenolics [26]. Mulinacci et al. compared the extraction time of phenolic compounds from strawberries with other extraction methods such as solid–liquid, subcritical water, and microwave-assisted method [27]. The results confirmed that UAE was the most effective method.

### 2.4. Techniques of Isolation and Purification of Bioactive Molecules from Plants

Purification and isolation of bioactive compounds from plants is a technique that has undergone new development in recent years [28,29]. This modern technique offers the ability to parallel the development and availability of many advanced bioassays on the one hand, and provided precise techniques of isolation, separation, and purification on the other. The goal when searching for bioactive compounds is to find an appropriate method that can screen the source material for bioactivity such as antioxidant, antibacterial, or cytotoxicity, combined with simplicity, specificity, and speed [27].

In vitro methods are usually more desirable than in vivo assays because animal experiments are expensive, take more time, and are prone to ethical controversies. There are some factors that make it impossible to find final procedures or protocols to isolate and characterize certain bioactive molecules. This could be due to different parts (tissues) in a plant, many of which will produce quite different compounds, in addition to the diverse chemical structures and physicochemical properties of the bioactive phytochemicals [30]. Both the selection and the collection of plant materials are considered primary steps to isolate and characterize a bioactive phytochemical. The next step involves a retrieval of ethno-botanical information to discern possible bioactive molecules. Extracts can then be made with various solvents to isolate and purify the active compounds that are responsible for the bioactivity. Column chromatographic techniques can be used for the isolation and purification of the bioactive compounds. Developed instruments such as High Pressure Liquid Chromatography (HPLC) accelerate the process of purification of the bioactive molecule. Different varieties of spectroscopic techniques like UV-visible, Infrared (IR), Nuclear Magnetic Resonance (NMR), and mass spectroscopy can identify the purified compounds [31].

### 2.5. Purification of the Bioactive Molecule

Many bioactive molecules have been isolated and purified by using paper thin-layer and column chromatographic methods. Column chromatography and thin-layer chromatography (TLC) are still mostly used due to their convenience, economy, and availability in various stationary phases [32]. Silica, alumina, cellulose, and polyamide exhibit the most value for separating the phytochemicals. Plant materials include high amounts of complex phytochemicals, which make a good separation difficult [32]. Therefore, increasing polarity using multiple mobile phases is useful for highly valued separations. Thin-layer chromatography has always been used to analyze the fractions of compounds by column chromatography. Silica gel column chromatography and thin-layer chromatography (TLC) have been used for separation of bioactive molecules with some analytical tools [32].

### 2.6. Structural Clarification of the Bioactive Molecules 

Determination of the structure of certain molecules uses data from a wide range of spectroscopic techniques such as UV-visible, Infrared (IR), Nuclear Magnetic Resonance (NMR), and mass spectroscopy. The basic principle of spectroscopy is passing electromagnetic radiation through an organic molecule that absorbs some of the radiation, but not all. By measuring the amount of absorption of electromagnetic radiation, a spectrum can be produced. The spectra are specific to certain bonds in a molecule. Depending on these spectra, the structure of the organic molecule can be identified. Scientists mainly use spectra produced from either three or four regions—Ultraviolet (UV), Visible, Infrared (IR), radio frequency, and electron beam [31]—for structural clarification.

### 2.7. UV-Visible Spectroscopy

UV-visible spectroscopy can be performed for qualitative analysis and for identification of certain classes of compounds in both pure and biological mixtures. Preferentially, UV-visible spectroscopy can be used for quantitative analysis because aromatic molecules are powerful chromophores in the UV range. Natural compounds can be determined by using UV-visible spectroscopy [33]. Phenolic compounds including anthocyanins, tannins, polymer dyes, and phenols form complexes with iron that have been detected by the ultraviolet/visible (UV-Vis) spectroscopy [34]. Moreover, spectroscopic UV-Vis techniques were found to be less selective and give information on the composition of the total polyphenol content. The UV-Vis spectroscopy was used to determine the total phenolic extract (280 nm), flavones (320 nm), phenolic acids (360 nm), and the total anthokyanids (520 nm). This technique is not time-consuming, and presents reduced cost compared to other techniques [35].

### 2.8. Infrared Spectroscopy

Some of the frequencies will be absorbed when infrared light passes through a sample of an organic compound; however, some frequencies will be transmitted through the sample without any absorption occurring. Infrared absorption is related to the vibrational changes that happen inside a molecule when it is exposed to infrared radiation. Therefore, infrared spectroscopy can essentially be described as a vibrational spectroscopy. Different bonds (C–C, C=C, C≡C, C–O, C=O, O–H, and N–H) have diverse vibrational frequencies. If these kinds of bonds are present in an organic molecule, they can be identified by detecting the characteristic frequency absorption band in the infrared spectrum [35]. Fourier Transform Infrared Spectroscopy (FTIR) is a high-resolution analytical tool to identify the chemical constituents and elucidate the structural compounds. FTIR offers a rapid and nondestructive investigation to fingerprint herbal extracts or powders.

### 2.9. Nuclear Magnetic Resonance Spectroscopy (NMR) 

NMR is primarily related to the magnetic properties of certain atomic nuclei; notably the nucleus of the hydrogen atom, the proton, the carbon, and an isotope of carbon. NMR spectroscopy has enabled many researchers to study molecules by recording the differences between the various magnetic nuclei, and thereby giving a clear picture of what the positions of these nuclei are in the molecule. Moreover, it will demonstrate which atoms are present in neighboring groups. Ultimately, it can conclude how many atoms are present in each of these environments [33]. Several attempts have been made in the past by using preparative or semi preparative thin-layer chromatography, liquid chromatography, and column chromatography to isolate individual phenols, the structures of which are determined subsequently by NMR off-line [34].

### 2.10. Mass Spectrometry for Chemical Compounds Identification 

Organic molecules are bombarded with either electrons or lasers in mass spectrometry and thereby converted to charged ions, which are highly energetic. A mass spectrum is a plot of the relative abundance of a fragmented ion against the ratio of mass/charge of these ions. Using mass spectrometry, relative molecular mass (molecular weight) can be determined with high accuracy and an exact molecular formula can be determined with a knowledge of places where the molecule has been fragmented [18]. In previous work, bioactive molecules from pith were isolated and purified by bioactivity-guided solvent extraction, column chromatography, and HPLC [36]. The techniques of UV-visible, IR, NMR, and mass spectroscopy were employed to characterize the structure of the bioactive molecule. Furthermore, molecules may be hydrolyzed and their derivatives characterized. Mass spectrometry provides abundant information for the structural elucidation of the compounds when tandem mass spectrometry (MS) is applied. Therefore, the combination of HPLC and MS facilitates rapid and accurate identification of chemical compounds in medicinal herbs, especially when a pure standard is unavailable [37,38,39,40]. Recently, LC/MS has been extensively used for the analysis of phenolic compounds. Electrospray ionization (ESI) is a preferred source due to its high ionization efficiency for phenolic compounds.

## 3. Lipid Oxidation

Lipid oxidation can occur during the processing, shipping, and storing of many foods. Lipids (such as triglycerides, sterols, and phospholipids) readily become oxidized with exposure to an oxidative environment [41]. Lipid molecules, especially those carrying polyunsaturated double bonds (i.e., linolenic acids), readily undergo oxidation within foods. Oxidatively stable oil with high melting temperature is necessary for solid fat application, and thus, highly saturated seed oil (palmitic acid and stearic acid) would be suitable for this end use [42]. Soybeans provide 56% of the world’s oilseed production. However, the percentage of saturated oil is very low in seed plants (about 10%), if compared to unsaturated oil (about 90%) [43]. Palmitic acid improves the oxidative stability of soybean oil, and can also be used to produce *trans*-fat-free shortening, margarine, and cosmetic products. However, this saturated short-chain fatty acid is undesirable for nutrition because its consumption results in an unfavorable lipoprotein profile in blood serum [44]. Stearic acid does not exhibit these cholesterolemic effects on human health [45]. Stearic acid is less likely to be incorporated into cholesterol esters and has a neutral effect on the concentration of blood serum LDL cholesterol [46,47]. Extensive research has been performed in order to increase stearic acid content oil production in the most widely consumed legume crop in the world, soybeans. By employing induced mutagenesis, seed stearic acid content was increased by up to 7 times [48].

Lipid oxidation in food systems can be caused by oxygen free radicals or reactive oxygen species. Free radicals are molecules with one or more unpaired electrons that work independently to cause oxidation [49]. Reactive oxygen species are a perfect example of oxygen free radicals. Reactive oxygen species do not solely contain free radical molecules, but also some non-free radicals that can influence lipid oxidation. Examples of non-free radical reactive oxygen species are hydrogen peroxide (H_2_O_2_), hydrochloric acid (HCl), ozone (O_3_), and molecular oxygen (O_2_) [42]. Molecular oxygen can react with linoleic acid about 1450 times faster than triplet oxygen. One of the major causes of oil rancidity is molecular oxygen. Lipid oxidation caused by the chain reaction of free radicals can be illustrated in three stages: initiation, propagation, and termination [42]:

(1) Initiation:
  RH + initiator → R
  ROOH + initiator → ROO•
(2) Propagation:
  R + O_2_ → ROO
  ROO + RH → ROOH + R•
(3) Termination:
  R + R → R-R
  ROO• + R → ROOH

The processes above occur in response to several physical or chemical factors including heating, radiation, temperature, metal ion catalysts, reactive oxygen species, and photosensitizers such as chlorophyll. The initiation step, shown in Equation (1), often happens at either an allytic methylene group of an unsaturated fatty acid (RH) or a lipid-hydroperoxide (ROOH). Next, the generated free radical (R•) reacts with oxygen to form a peroxy radical (ROO•). This product can directly react with another lipid molecule to produce a lipid hydroperoxide (ROOH), and thus a lipid free radical (R•). This causes continuously cascading chain reactions to occur until the free radicals are neutralized by other free radicals. This whole stage is shown in Equation (2). In the termination step, there are two radicals that have converted into non-free radical products, and thus will stop the cascade mode of the chain reaction according to Equation (3). Moreover, the reaction chain can also be terminated by some antioxidants or free radical scavengers. Metal ions, especially those of iron and copper, effectively catalyze these reactions [50].

Lipoxygenases (EC 1.13.11) can also act, causing oxidation to produce the peroxides in food materials that contain lipids. Hydrogen peroxide is one of the primary products of the oxidation, and it is very unstable and easily converts into secondary products. The final product of oxidation may include different chemical groups such as aldehydes, ketones, alcohols, acids, or hydrocarbons. These kinds of compounds can have a negative effect on the appearance, quality, and edibility of a food product by changing the texture, color, flavor, and safety of foods, or also by producing unacceptable off odors or off tastes, even negatively affecting the nutritional value [50].

## 4. Plants as a Source of Antioxidants

Antioxidants can be defined as bioactive compounds that inhibit or delay the oxidation of molecules [42]. Antioxidants are categorized as natural or synthetic antioxidants. Some synthetic antioxidants commonly used are: BHT, BHA, propyl gallate, and tertbutylhydroquinine. Many scientists have concerns about safety because synthetic antioxidants have recently been shown to cause health problems such as liver damage, due to their toxicity and carcinogenicity. Therefore, the development of safer antioxidants from natural sources has increased, and plants have been used as a good source of traditional medicines to treat different diseases. Many of these medicinal plants are indeed good sources of phytochemicals that possess antioxidant activities. Some typical examples of common ingredients that have been used in ethnic foods are tamarind, cardamom, lemon grass, and galangal basil. These spices or herbs have been shown to contain antioxidants [51].

Deterioration of food due to either bacterial or fungal infection has always been a major concern, causing huge losses to food industries and societies throughout the world [51]. Moreover, the spread of food pathogens has become a major public health concern. With an increasing awareness of the negative effects of synthetic preservatives, there has been increased demand for the use of nontoxic, natural preservatives, many of which are likely to have either antioxidant or antimicrobial activities [52,53]. Herbs have always been used for flavor and fragrance in the food industry, and some of them have been found to exhibit antimicrobial properties [54]. Therefore, the call for screening and using plant materials for their antioxidant and antimicrobial properties has increased. Approximately 20% of all plant species have been tested in both pharmacological and biological applications to confirm their safety and advantages [3]. A summary of the types of compounds, plant species, plant parts from which compounds were extracted, etc. can be found in Table S1.

### 4.1. Presence of Antioxidant in Red Algae

Red algae are aquatic plant species considered one of the oldest groups of eukaryotic algae [55]. The antioxidant activity of a red alga, *Palmaria palmate*, has been studied. The results reported that 9.68 μg of ascorbic acid and 10.3 μg of total polyphenol can equally reduce activity in 1 mg of dulse extracts. The reducing activity was correlated with aqueous/alcohol soluble compounds due to the presence of functional groups such as hydroxyl, carbonyl, etc., which lead to reduced or inhibited oxidation [56].

### 4.2. Antioxidants from Monocots

Ashawat et al. studied the antioxidant properties of ethanolic extracts of *Areca catechu* and showed that *Areca catechu* had the highest antioxidant activity when compared to other eudicots like *Centella asiatica, Punica granatum*, and *Glycyrrhiza* *glabra* [57]. Londonkar and kamble studied *Pandanus odoratissimus* L. in order to determine its antioxidant activity [58]. Zahin et al. screened *Acorus calamus* to estimate antioxidant activity and total phenolic contents [59]. The observations confirmed that there was a significant correlation between the phenolic content and antioxidant activity. Another monocot, *O. sanctum*, showed that the inhibition of lipid peroxidation in vivo and in vitro increased proportionally with an increase in the concentration of the extract.

### 4.3. Antioxidants from Vegetables

Consumption of vegetables has been linked to a reduction in the risk of many diseases, such as cancer and cardiovascular disease, when studied in epidemiological studies [59]. Numerous studies have attempted to screen vegetables for antioxidant activity by using different oxidation systems. These vegetables include carrots, potatoes, sweet potatoes, red beets, cabbage, Brussels sprouts, broccoli, lettuce, spinach, onions, and tomatoes. In addition to the concise studies, which have used different methodologies to release bioactive compounds, it is becoming increasingly difficult to ignore advanced extraction methods, which have paved the way to extract bioactive compounds rapidly. Despite scientists’ successes in showing the activity of vegetables’ bioactive compounds, there is little known about the activity of the antioxidant components that have been isolated from these vegetables. Researchers have tended to focus on advanced methods to isolate and measure the activity of antioxidant compounds such as flavonoids, phenolic acids, tocopherols, carotenoids, and ascorbic acid [60].

### 4.4. Antioxidants from Fruits

Fruit consumption has also been linked to a reduction in the risk of many diseases [61]. Peaches (*Prunus persica* L.) are an economically important fruit in many countries. Studies have shown that phenolic compounds found within various peach genotypes are a major source of potential antioxidants [60]. Interestingly, peaches have shown a great inhibition of low density lipoprotein (LDL) oxidation with a percentage of antioxidant activity of 56–87%. This antioxidant activity can be attributed to its essential compound content including hydroxycinnamic acids, chlorogenic, and neochlorogenic acids, but not to carotenoids such as b-carotene and b-cryptoxanthin. Moreover, low antioxidant activity was found in peach peel. In contrast, Plumb et al. pointed out that hydroxycinnamic acids do not contribute to the inhibition of lipid peroxidation of the liver using plums and peaches because hydroxycinnamic acids had weak ability to scavenge hydroxyl radicals [62].

Grape (*Vitis vinifera* L.) is a fruit crop grown throughout the world. Grapes and its juices have been recently studied by [62]. Phenolic compounds were high in both fresh grapes and commercial grape juices. The percentage of inhibition LDL oxidation was about 22% to 60% for fresh grapes, while it was approximately 68% to 75% for commercial grape juices, when standardized at 10 mg gallic acid equivalents (GAE). According to [63], both grapes and its juices exhibited high oxygen radical absorbance capacity (ORAC), and the anthocyanin pigment malvidin-3,5-diglucoside was a major compound isolated in grapes. Anthocyanins with malvidin nucleus malvidin 3-*O*-(6-*O*-p-coumaroylglucosido)-5-glucoside and phenolics were common compounds isolated from wild grapes (*Vitis coignetiae*). Wangensteen et al. tested the activity of many bioactive compounds by releasing them from grape pomace, and demonstrated that bioactive compounds have the ability to significantly inhibit LDL oxidation in the human body [64]. Grape seeds are an amazing source of polyphenol compounds including monomerics such as catechin, epicatechin, and gallic acid, and polymerics such as procyanidins [65].

Both polyphenols and carotenoids are the major phenolic compounds of apples (*Malus domestica* L.) including caffeic, quinic, and p-coumaric acids. These polyphenols can act as antioxidants. Flavanol monomers and oligomers, as well as quercetin, contribute to the beneficial health aspects of fruits and vegetables [65]. Apple pomace has mainly been used as a major source of polyphenols such as chlorogenic acid [66,67]. In addition phenolics like caffeic, p-coumaroyl quinic, arbutin, p-coumaric acids, and especially flavonol procyanidins have been mentioned as constituents of apple pomace [68]. The ability of procyanidins to work as oxygen radical scavengers, superoxides, and hydroxyl radicals was estimated. Despite the low content in total phenols in apples obtained by using acetone 70%, it has shown strong antioxidant activities towards oxidation of linoleic acid. In this case, the major bioactive compounds obtained were chlorogenic acid and phloretin glycosides; however, Vitamin C was a minor fraction in apple juice [69].

Antioxidant and antibacterial activities of various solvent (ethyl acetate, acetone, methanol, and water) extracts of *Punica granatum* peel were examined by applying the 2,2-diphenyl-1-picrylhydrazyl (DPPH) radical scavenging method. The results obtained showed a significantly higher decreasing power in the methanol extracts and a significantly higher antibacterial activity in the acetone extracts.

Soong and Barlow investigated the antioxidant activity and phenolic content of various fruit seeds [70]. Petroleum ether was used to get rid of the excess fat from the seeds and extraction has been carried out with methanol. The 2,2-azino-bis-3-ethylbenzthiazoline-6-sulfonic acid (ABTS), DPPH, and the ferric reducing ability of plasma (FRAP) methods were used to investigate the antioxidant activity. Abdille et al. examined the antioxidant activity of *Dillenia indica* fruit using different kinds of solvents using DPPH, phospho-molybdenum, and β carotene bleaching methods [71]. The methanol extracts showed the highest antioxidant activity, followed by the ethyl acetate and water extracts. Antioxidant activity of *Syzygium cumini* fruit in vitro has been investigated [71]. Antioxidant activity was measured by DPPH, superoxide, lipid peroxidation, and hydroxyl radical scavenging activity methods. The results brought to light a significant correlation between the concentration of the extract and the percentage of inhibition of free radicals. The antioxidant property of the fruit might be from the presence of antioxidant vitamins, anthocyanins, phenolics, and tannins. It has been reported that blackberry (*Rubus fruticosus* L.) fruit extracts produced in varying climatic regions showed that antioxidant activity depended on the genotype, rather than the climate or season [10]. Juntachote and Berghofer measured the stability of the antioxidant activity of ethanolic extracts for Holy basil and galangal using DPPH, superoxide, β carotene bleaching, reducing power, and iron chelation methods [72]. They found higher antioxidant activity at neutral pH compared to an acidic pH. Holy basil and galangal extracts provided strong iron chelation activity, superoxide anion scavenging activity, and reducing power proportional to the concentration of the extracts. Liyana-Pathirana et al. investigated the antioxidant activity of cherry laurel fruit (*Laurocerasus officinalis* Roem) and its concentrated juice (Pekmez) using in vitro methods such as superoxide, DPPH scavenging activity, and inhibition of LDL oxidation [73]. The results confirmed the presence of a significantly higher antioxidant activity in pekmez compared to the cherry laurel fruit. Employing in vitro methods such as DPPH and superoxide scavenging activity, Orhan et al. measured the antioxidant activity of *Arnebia densiflora* Ledeb and observed that polar extracts had a higher antioxidant activity compared to non-polar extracts [74]. Rathee et al. studied the antioxidant activity of *Mammea longifolia* buds extracted in both methanol and aqueous ethanol. The results found a significant antioxidant activity, and the activity of aqueous ethanol was higher than methanol. The antioxidant activity of leaf extracts of *Annona* species in vitro reveals that *Annona muricata* possessed a higher antioxidant activity compared to *Annona squomosa* [75].

### 4.5. Cooking Herbs as an Important Source of Antioxidants

The antioxidant activity of 32 herbs belonging to 21 different families has been screened [76]. The finding confirmed that there was a positive correlation between the total antioxidant activity and total phenolic content. Lu and Yeap Foo studied *Salvia officinalis* (L.) for its antioxidant activity and polyphenol content and reported that rosmarinic acid and various catechols were responsible for the radical scavenging activity and caffeic acid was responsible for the xanthine oxidase (EC 1.17.3.2) inhibition [77]. Zhao et al. investigated the antioxidant activity of *Salvia miltiorrhiza* and *Panax notogensing* [78]. The results showed that *Salvia miltiorrhiza* had a higher reducing power and scavenging activities against free radicals, including superoxide and hydroxyl radicals, although it showed weak hydrogen peroxide scavenging.

Furthermore, Javanmardi et al. tested the Iranian *Ocimum* sp. accessions to determine the antioxidant activities and total phenolic contents and demonstrated that the antioxidant activity increased in parallel with the total phenolic content [51].

Evaluation of the pomegranate peel extracts to discover its antioxidant and antimutagenic activities using different solvents such as ethyl acetate, acetone, methanol and water has been carried out [51]. Dried extracts were examined by using the Ames test and the phosphorus-molybdenum method to test both anti-mutagenic and antioxidant activities. The results showed the highest anti-mutagenic and the lowest antioxidant activity in the water extract.

Moreover, the phenolic content and antioxidant activity of parsley (*Petroselinum crispum*) and cilantro (*Coriandrum sativum*) have been tested [79]. The total phenolic content was observed to be different between parsley and cilantro leaves and stems, as well as methanol and water extracts. The methanol leaf extracts exhibited significant antioxidant activity towards both lipid- and water-soluble radicals. The works also investigated the antioxidant activity of aqueous plant extracts using in vitro methods such as DPPH scavenging activity and FRAP. The results revealed a strong correlation between total antioxidant activity and phenolic content and a weak correlation between cupric ion chelators and polyphenols. The antioxidant activity and lipid peroxidation inhibition of *Satureja montana* L. subsp. *Kitaibelii* extracts were tested using hydroxyl radical scavenging. The results obtained showed that there was a significant correlation with total phenolic content [9].

### 4.6. Antioxidant from Legumes

Antioxidant property of methanol extracts of *Mucuna pruriens* L. (*Fabaceae*) seed extracts has been investigated in vitro using the DPPH radical scavenging method. The results obtained showed a positive correlation between the antioxidant activity and the total phenolic compounds [80]. Siddhuraju and Manian studied horsegram (*Macrotyloma uniflorum* Lam.) seeds to measure the antioxidant and free radical scavenging activity [81]. Acetone extracts had a higher activity of about 70% [81]. Samak et al. studied *Wagatea* sp. to measure its scavenging activities of superoxide and hydroxyl radicals and showed a high oxidation inhibition because it was rich in both phenolic and flavonoid contents. The authors also reported that bark and leaf extracts of *Wagatea* sp. exhibited high scavenging action against super radicals [82].

### 4.7. Antioxidants from Trees

Antioxidants from trees have been also measured. Phenolics from almond hulls (*Prunus amygdalus* L.) and pine sawdust (*Pinus pinaster* L.) have been extracted employing various methods in order to determine the gram fresh yield of polyphenol compounds and antioxidant activity [83]. The antioxidant activity was measured by the DPPH radical scavenging method. The results showed that ethanol was most appropriate either for phenolics or any bioactive compounds, while methanol was more selective for extracting polyphenolics. The antioxidant activity of juniper (*Juniperus communes*) fruit extracts has been investigated in vitro [84]. The results confirmed that both water and ethanol extract showed strong antioxidant activity. The concentration of 60 μg/mL of water and ethanol extracts exhibited 84% and 92% inhibition, respectively, on the peroxidation of linoleic acid. Ibrahim et al. studied the antioxidant activity of *Cupressus sempervirens* L., and set up goals to isolate quercetin, rutin, cupress flavone, caffeic acid, and para-coumaric acid. The results showed higher antioxidant activity related to quench DPPH and identified these active compounds successively [85].

Higher values of antioxidant activity have been obtained by using a methanolic solvent to extract the bioactive compounds from *Anacardium occidentale*, while other solvents like ethyl acetate gave lower values of antioxidant activity [85]. Kaur et al. studied the Chickrassy *Chukrasia tabularis* A. Juss leaves to confirm its ability to inhibit lipid peroxidation and showed that there was a large inhibition considering its high content of phenolic compounds [86]. Finally, *Acacia nilotica* L. antioxidant activity has been measured using ethyl acetate as a solvent to extract phenolic compounds [86]. The results exhibited the highest antioxidant activity when the concentration of extracts was relatively high.

### 4.8. Antioxidant from Shrubs

Many shrubs have been shown to contain antioxidant activity. Singh et al. tested several plants to measure the antioxidant activity from different extracts. The antioxidant activity was determined by using peroxide value, thiobarbituric acid, DPPH radical scavenging activity, and reducing power. The results showed that the antioxidant activity of *Coriandrum sativum* L. and *Sarcolobus globosus* L. exhibited high activity by using acetone solvent, and its activity was similar to synthetic antioxidants [87].

Eleven Algerian medicinal plants have been measured for phenolic compound content and antioxidant activity using the ABTS method. The tested plants showed antioxidant activity. *Artemisia campestris* L. had better antioxidant activity than caffeic acid and tocopherol. Moreover, HPLC analyses exhibited a good correlation between the antioxidant activity and hydroxycinnamic derivative content.

Evaluation of *Vitex negundo Linn* seed antioxidant activity using different methods such as superoxide, hydroxyl, and DPPH scavenging activity has been carried out [87]. The highest antioxidant activity was in both raw and dry heated seed extracts, while lower antioxidant activity was observed in the hydrothermally processed samples.

### 4.9. Characterization of Antioxidants from Other Eudicots

The nitric oxide and superoxide scavenging activity of green tea have been studied by Nakagawa and Yokozawa [88], who concluded that certain tannins had the ability to exhibit excellent antioxidant activity. Zin et al. estimated the antioxidant activity of the extracts from various parts of Mengkudu (*Morinda citrifolia* L.), including the leaves, fruits, and roots, using different solvents such as methanol and ethyl acetate [89]. Ferric thiocyanate and thiobarbituric acid were used as models to observe and evaluate the antioxidant activity. The results exhibited a higher antioxidant activity in the methanol extract of Mengkudu root, although it was not significantly different from tocopherol and BHT extracts. The methanol extracts of the fruits and leaves showed unassuming activity. According to these scientists, the antioxidant activity in the roots resulted from polar and non-polar compounds, but the antioxidant activity in leaves and fruits was only due to non-polar compounds.

Increase of the antioxidant activity of fennel (*Foeniculum vulgare*) seed extracts in vitro has been shown to be proportional to the increase in the concentration of extract [89]. Nine other extracts of Bolivian plants have been measured for radical scavenging and antioxidant activity using the DPPH and β carotene bleaching methods [90]. It was found that the ethyl acetate fractions had higher radical scavenging and antioxidant activity compared to the other extracts. It has been reported that the bioactive compounds of *Rhodiola rosea* extracted in methanol showed a significant yield of phenolics, about (153 ± 2 mg/g) [91]. Wangensteen et al. investigated the antioxidant activity of *Ss globosus* using DPPH scavenging and inhibition of lipoxygenase [64]. Coriander had a high capacity to inhibit oxidation. There was also a positive correlation between total phenolics and antioxidant activity. Moreover, it was observed that the leaves of the coriander had higher antioxidant activity than the seeds [91].

Antioxidant activity of *Phyllanthus niruri* was estimated using methanol and water as a solvent. The extracts of leaves and fruits exhibited high antioxidant activity by using the inhibition of lipid peroxidation and DPPH scavenging [64]. The results also noticed a higher superoxide scavenging activity in the aqueous extract compared to the methanol extract. Moreover, the antioxidant and free radical scavenging activity of *Phyllantus* species from India in an aqueous extract has been also evaluated [92]. The antioxidant activity was estimated using DPPH, β carotene, superoxide, nitric oxide scavenging, and reducing power methods. The extract of *Coleus aromaticus* exhibited a moderate inhibition on DPPH and nitric oxide scavenging activity. 

*Panax* exhibited strong iron chelating and weak superoxide scavenging. Ajila et al. carried out bioactive compounds and antioxidant activity of mango peel extract [93]. The results showed a higher concentration of anthocyanins and carotenoids in the ripe peel compared to the raw peel, while the raw peel exhibited higher polyphenol content. The range of IC50 values of lipid peroxidation and DPPH were 1.39–5.24 μg of gallic acid equivalent. Chen and Yen investigated the antioxidant activity and free radical scavenging capacity of dried guava leaves and fruit [94]. The results confirmed that guava leaf and guava tea extracts had the ability to inhibit oxidation by 94–96% at a concentration of 100 μg/mL. Fruit extracts exhibited less activity compared to leaf extracts, while the scavenging effect increased with an increase in the concentration. Also, there was a correlation between antioxidant activity and phenolic compounds. Dastmalchi et al. investigated the chemical composition and antioxidant activity of water-soluble Moldavian balm (*Dracocephalum moldavica*) in vitro by using DPPH, ABTS, and superoxide activity [95]. The finding confirmed that polar compounds such as caffeic acid and rosmaric acid were responsible for the antioxidant activity observed.

Mulberry leaves were investigated to determine the antioxidant activity using different solvents [95]. The procedure used DPPH and inhibition of lipid peroxidation methods to evaluate its activity. The results showed that the methanolic extract exhibited the highest yield of total phenolics, and it was the most essential antioxidant in all the methods used. The antioxidant activity of kale (*Brassica obraceae* L.) has been screened after removing a fat fraction from the samples [96]. The extraction process used methanol to investigate its antioxidant activity while using DPPH scavenging activity as tested method. The works successfully isolated nine phenolic acids using HPLC and MS, and confirmed that the total phenolic content was correlated with DPPH scavenging activity.

In another study, ethanol has been used to estimate the antioxidant activity of sun-dried cashew nuts (*Anacardium occidentale* L.) skin [97]. First, bioactive compounds were extracted with a protocol including lipid peroxidation, ABTS, and DPPH methods to measure the capability to inhibit oxidation. The results found that epicatechin was the major polyphenol in the extract, which was responsible for antioxidant activity.

Kaviarasan et al. measured the antioxidant and antiradical activity of fenugreek (*Trigonella foenum* ssp. *graecum*) seeds in vitro; the results showed that there was a positive relationship between the antiradical activity and phenolic compound content in the extract [98]. Hexane and methanol were used to extract the bioactive compounds and measured the antioxidant activity of *Pueraria tuberosa* by using ABTS, lipid peroxidation, and superoxide and hydroxyl scavenging activity. An independent study has shown an inhibition of the lipid peroxidation [99].

The rhizome of the lotus (*Nelumbo nucifera* Gaertn.) has been measured for its antioxidant activity in various solvent extracts using β Carotene bleaching and DPPH methods [99]. Methanol extraction had a higher DPPH scavenging activity than acetone. *Helichrysum pedunculatum* has been tested to determine the antioxidant activity, and total phenolic and flavonoid content [100]. The results demonstrated that whenever the amount of phenolic content and flavonoid content was increased, higher antioxidant activity was obtained. Meot-Duros and Magn screened the leaves of *Crithmum maritmum* to show if there was any correlation between the antioxidant activity and phenolic content and found a significant correlation between antioxidant activity and phenolic content when methanol was used as the solvent [101].

Another dicot, *Tricholepis glaberrima* L. (*Asteraceae*), has been investigated for antioxidant activity using different kinds of extracts [101]. Higher antioxidant activity was found by methanol, and a lower antioxidant activity in both chloroform and aqueous extracts. Sakat et al. investigated *Oxalis corniculata* L. in order to measure the antioxidant and anti-inflammatory activity employing methanol as a solvent. The IC 50 values of DPPH and nitric acid were about 93 and 73.07 μg/mL, respectively [102].

Jain et al. studied *Tabernaemontana divaricata* L. to determine the phytochemical and free radical scavenging activities in vitro. The results indicated that the antioxidant activity was the same in both ethanol and water extracts, but less in petroleum ether [103].

It has been reported that *Ascleipiadaceae* and *Periplocoideae* presented high antioxidant activity, with the presence of a strong correlation between antioxidant activity and phenolic content [103]. Laitonjam and Kongbrailatpam studied the chemical composition and antioxidant activities of *Smilax lanceafolia* by isolating the flavonol glycoside and steroidal saponin, which showed high antioxidant activity [104]. Spinach (*Spinacea olerace* L.) is among the most popular vegetables in the world. It was domesticated and first cultivated in West Asia. According to analytical chemistry, spinach is a source of violaxanthin and neoxanthin antioxidants that cannot be commercially produced [105]. Although they may be present, pigments such as carotenoids can be masked by chlorophyll in greenish vegetables such as spinach [106]. B-carotene, lutein, violaxanthin, and neoxanthin are the major carotenoids in raw spinach [107]. Pumpkins belong to the family *Cucurbitaceae*. This family is classified depending on the texture and shape of stems, such as in *Cucurbita pepo*, *Cucurbita moschata*, *Cucurbita maxima*, and *Cucurbita mixta*. Nowadays, the market offers a wide variety of vegetables, with pumpkin being one of them because of its many applications for nutrition or decoration [108].

## 5. Plants Vitamins and Phenolic Compounds as Antioxidants

### 5.1. Phenolic Compounds

#### 5.1.1. Phenols and Phenolic Acid

**Figure 1:**
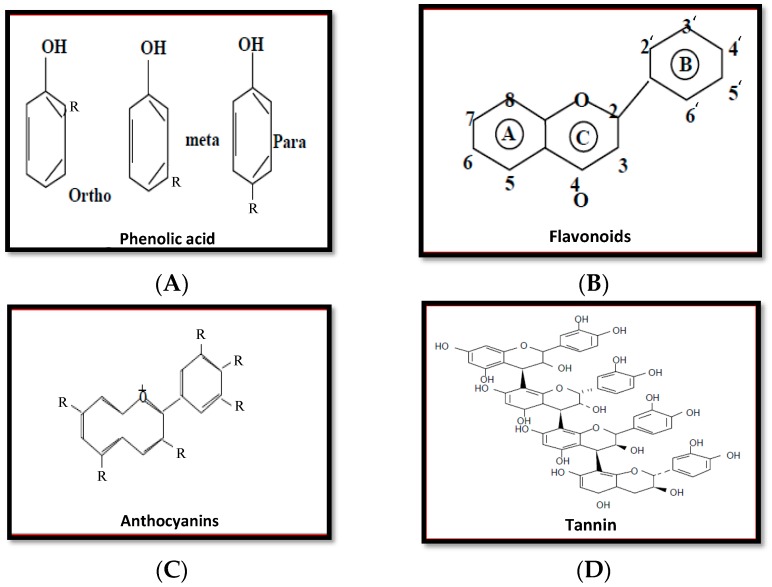
Chemical structure of phenolic acid (**A**), flavonoids (**B**), anthocyanins (**C**), and tannins (**D**).

Phenolic acids contain carboxylic acid in the chemical composition. Hydroxycinnamic and hydroxybenzoic acids are both main pillars of phenolic acids, according to Figure 1A. Moreover, scientists have noted that p-coumaric, caffeic, ferulic, and sinapic acids are main components of the hydroxycinnamic acids (Figure 1A).

#### 5.1.2. Flavonoids

Flavonoids have a low molecular weight (Figure 1B). Flavane is an example of a flavonoid. Flavane contains two benzene rings (Figure 1A,B) within its chemical composition. These two rings connect to each other through a pyrane ring (Figure 1C). Flavones, isoflavones, flavonoids, flavonols, flavanones, anthocyanins, and proanthocyanidins are part of flavonoids according to the flavonoid classification (Figure 1B).

#### 5.1.3. Anthocyanins

Anthocyanidins are a simple example of anthocyanins. Anthocyanidins consist of an aromatic ring that is linked to a heterocyclic ring (Figure 1C). Moreover, the heterocyclic ring is connected to the third aromatic ring through a carbon bond [109]. Scientists have noted that anthocyanins are often found in a glycoside form. Moreover, many kinds of anthocyanins are found in nature, making these kinds of phenolic compounds very complex. Scientists have noted that anthocyanins in different kinds of fruit are considered an essential compound that can enrich and increase antioxidant activity (Figure 1C).

#### 5.1.4. Tannins

Tannins are natural products present in several plant families, and have large amounts of phenolic rings in the structure. Tannins are classified into two groups: hydrolyzable and condensed. Condensed tannins contain flavonoids units with several degrees of condensation. Hydrolyzable tannins are considered a mixture of simple phenols with ester linkages in its structure. There are many factors such as alkaline compounds, mineral acids, and enzymes that have the ability to hydrolyze tannins (Figure 1D) [110].

### 5.2. Vitamins Role in Cancer Prevention

Cancer has been increasing throughout the world. It is the main cause of mortality from year to year. There were 10.4 million new cancer cases registered in 2015, and scientists predict that the number of cancer cases per year will double by 2030 [111]. Recently, many studies have shown rigorous evidence that hydroxyl radicals (OH•) and the superoxide anion ($O_{2}^{-}$•) are involved in the development of cancer because they are biological reactive oxygen species. Compounds with high reactive oxygen species reduction activity are likely able to prevent cancer’s occurrence [112]. As shown previously, fruits and vegetables are the primary source of natural antioxidants, consisting of different kinds of antioxidant compounds such as Vitamin C, Vitamin E, carotenoids, lutein, and lycopene. Some researchers have confirmed that phenolic compounds and polyphenols are secondary plant metabolites, which are considered the best scavengers to prevent the production of free radicals. The United States has an amazing diversity of plant species. Some of them have been used for traditional medicines for a long period of time because of their various desirable activities. Kiwi and pomegranate plants extracts were screened to show the cytotoxic effects on two tumor cell lines (*L20B* and *RD*). The results have shown that the means of both L20B and RD cultures were significantly different (*p* < 0.05), and kiwi and pomegranate plant extracts exhibited a strong ability to inhibit the growth rate of L20B and RD cell lines. At concentrations of 1000 μg/mL, both extracts showed a high ability to decrease the number of L20B and RD cells when compared with the control [113].

The mixtures of the plant natural products have been screened in order to study their effect on human leukemia cells [114]. The finding confirmed that mixtures of natural products were a good source for human leukemia cell inhibition. Nassr-allah et al. investigated the chemical diversity of natural products from plants in order to test their ability to work as anticancer and antioxidant agents [115]. DPPH assay was used to measure the antioxidant activity for plant extraction while using in vivo and vitro methods in order to measure the anticancer activity. The results confirmed that some natural products from Egyptian flora have the potential for use as therapeutics for diseases such as cancer [116].

The effectiveness of an aqueous extract from willow leaves (*Salix safsaf*, *Salicaceae*) against human carcinoma cells has been tested in vivo and in vitro [115]. The findings mentioned that the metabolites for the willow extract could inhibit tumors, thereby enhancing apoptosis and causing DNA damage. The anticancer activity of different extracts from the leaves of the drumstick tree (*Moringa oleifera*) was screened in order to test against leukemia and hepatocarcinoma cells in vitro. Primary cells harvested from 10 patients with acute lymphoblastic leukemia (ALL) and 15 with acute myeloid leukemia (AML) were significantly killed by hot water and ethanol extracts. Thus, *Moringa oleifera* may have the potential for use as a natural treatment for diseases such as cancer [117]. Altemimi reported that the phenolic extracts from the olive leaf extract could be used as a source of potential antioxidant and antimicrobial agents [118].

## 6. Plants as an Antimicrobial Source

The antibacterial activity of *Punica granatum* extracts has been investigated by using various solvents [119]. The water extract had the ability to inhibit *Bacillus subtilis* and *Staphylococcus aureus*, but the organic solvents have the ability to inhibit the growth of all the organisms tested. Shariff et al. estimated the antibacterial activity of *Rauvolfia tetraphylla* and *Physalis minima* leaves. The chloroform extract was a more powerful inhibitor of pathogenic bacteria [120].

Indian medicinal plants have been shown to have antimicrobial activity [120]. About 77 extracts belonging to these plants have been tested for their antimicrobial ability against eight species of bacteria and four species of pathogenic fungi. The findings showed that water extracts of *Lantana camara* L., *Saraca asoca* L., *Acacia nilotica* L., and *Justicia zeylanica* L. caused the highest growth inhibition of all tested bacteria. The antimicrobial activity was the highest, ranging between 9.375 and 37.5 μg/mL and 75.0 to 300.0 μg/mL against both bacterial and fungal pathogens.

Devi et al. investigated *Achyranthes bidentata* Blume to determine its phytochemical content and antibacterial activity [121]. The antibacterial ability of the ethanol extract effectively inhibited *Bacillus subtilis*, *Salmonella typhi*, and *Klebsiella pneumoniae*, but was less effective against *Pseudomonas* species and *Staphylococcus aureus* [122]. Ethanolic extracts of *Gymnema montanum* L. have been studied to measure its antimicrobial properties against *Salmonella typhi*, *Pseudomonas aeruginosa*, and *Candida albicans* [121]. The results indicated the highest presence of antimicrobial properties in the leaf extract of *G. montanum,* correlating to its phenolic compound content. The antimicrobial activity of *Piper ribesoides* L. from methanolic root extract against *Staphylococcus aureus* has been reported [123]. Interestingly, a small amount of 3.125 mg/mL was enough to inhibit harmful bacteria. Leaf extracts of *Caesalpinnia pulcherrimma* (L.) showed higher antioxidant activity in water and ethanol extracts and lower antioxidant activity in petroleum ether extracts [124]. *Torilis japonica* L. fruit has been observed to reduce the amount of spores, and the concentration of the vegetative cell was lower than the detection level. Ghosh et al. studied *Stevia rebaudiana* Bertoni to measure its antimicrobial properties against 10 pathogens [125]. The findings confirmed that *Staphylococcus aureus* was more susceptible than others [24]. Mahesh and Satish screened some important medicinal plants to show the antibacterial activity on human pathogenic bacteria [126]. Water and methanol were used as solvents to extract the phenolic compounds. The finding confirmed that the methanol extract had a higher antimicrobial activity than the aqueous extract [125].

Moreover, leaf extracts of *Acacia nilotica* L., *Sida cordifolia* L., *Tinospora cordifolia* L., *Withania somnifera* L., and *Ziziphus mauritiana* L. have been studied to determine the antibacterial activity against *Bacillus subtlis*, *Escherichia coli*, *Staphylococus aureus*, and *Pseudomonas fluorescens*, as well as studying the antifungal activity against *Aspergillus flavus*, *Dreschlera turcica*, and *Fusarium verticilloides* [126]. The highest antibacterial activity was noticed in *Acacia nilotica* and *Sida cordifoliain* leaves, and the highest antifungal activity was noticed in *Acacia nilotica* bark. Water and methanol extracts of *Samanea saman* (Jacq.) exhibited a significant effect against *Xanthomonas* spp. and human pathogenic bacteria. *Pseudarthria viscida* root has been studied to measure its antimicrobial activity using ethanol as a solvent. The results showed high antimicrobial activity when compared to standard drugs like ciprofloxacin and griseofulvin.

Ehsan et al. reported a high antimicrobial activity against *Staphylococcus aureus* using methanol and ethanol extracts for *Hopea pariviflora* Beddome [127]. Ethanolic extracts of *Bryonopsis laciniosa* have been investigated for their antimicrobial activity against different Gram-positive and Gram-negative bacteria. The growth of *Staphylococcus aureus*, *Micrococus luteus*, and *Bacillus cereus* was inhibited, as shown by a decrease in the growth zone.

*Plumbago zeylanica* L. has been screened to measure the antibacterial activity in chloroform extracts to show antimicrobial activity against *Escherichia coli*, *S. typhi*, and *Staphylococcus aureus* [127]. However, *Bacillus subtilis* and *Klebsiella* were resistant. Khond et al. studied 55 medicinal plants to measure the antimicrobial activity [128]. The higher antibacterial activities were in the extracts of *Madhuca longifolia* L., *Parkia biglandulosa* L., and *Pterospermum acerifolium* L. compared to the other plants screened. Pavithra et al. screened *Evolvulus nummularius* L. for its antibacterial activity, finding that *Escherichia coli* and *B. subtilis* were the most inhibited by an ethanolic extract [129].

*Hygrophila spinosa* Andres leaves showed significant antibacterial activity when collected between September to October, with less activity seen during other months [129]. *Artemisia pallens* L. has been studied for its antimicrobial activity against seven species of bacteria [130]. The results found that *Bacillus cereus* was more sensitive to *A. pallens* extracts. Also, a methanolic extract exhibited higher antibacterial activity than the other solvents used. Akroum indicated the antimicrobial activity of some Algerian plants [131]. The results expressed higher antibacterial activity in methanolic extracts of *Linum capitatum*, *Camellia sinensis*, *Allium schoenoprasum*, *Vicia faba*, *Citrus paradise*, *Lippia citriodora*, *Vaccinium macrocarpon*, and *Punica granatum*. Bajpai et al. screened the antibacterial activity of *Pongamia pinnata* leaves by using methanol and ethyl acetate extracts to confirm its ability against certain pathogenic bacteria [132]. The results exhibited significant inhibition compared to streptomycin. It has been demonstrated that *Memecylon edule* has higher antibacterial activity in chloroform extracts compared to other extracts [132]. Gram-negative bacteria were more susceptible to the crude extracts compared to Gram-positive bacteria. Bansal et al. studied plants found in arid zones in order to determine the antibacterial efficiency [133]. An ethanolic extract of *Tinospora cordifolia* L. inhibited *Bacillus cereus* and *Staphylococcus aureus*. Kumar et al. reported *Andrographis serpyllifolia* L. to have significant antimicrobial activity against tested organisms in methanol extracts of both aerial parts and root [134].

*Memecylon malabaricum*, *Cochlospermum religiosum,* and *Andrographis serpyllifolia* have been rested for their possible antimicrobial activity [135,136]. Moderate activity against both Gram-positive and Gram-negative bacteria was observed. The antimicrobial activity of an ethanolic extract of *Anethum graveolens* was better than the aqueous extract. Khanahmadi et al. [137] found a higher antibacterial activity against Gram-positive bacteria compared to Gram-negative bacteria when an ethanolic extract of *Smyrnium cordifolium* Boiss was used [136,137]. Koperuncholan et al. studied some medicinal plants of the south eastern slopes of the Western Ghats [138]. Gram-positive bacteria were more sensitive than Gram-negative bacteria to the plant extracts. Niranjan et al. screened *Schrebera swietenioides* Roxb to measure the effectiveness against human pathogenic bacteria [139]. Water and methanol extracts were most effective to prohibit growth of all the harmful bacteria tested.

Different studies have isolated tannins and saponins from some Indian medicinal plants, testing the antibacterial activity against *Klebsiella pneumoniae* [139,140]. Ethanol extracts of *Tinospora cordifolia* strongly inhibited *Bacillus cereus* and *Staphylococcus aureus*. Also, significant antibacterial activity from ethanolic extracts of *Coleus aromaticus* L. has been found. The most effective range of inhibition was at concentrations of 25–39 μg/mL. Vinothkumar et al. evaluated a *Andrographis paniculata* L. leaf extract’s ability to inhibit the growth of Gram-positive and Gram-negative bacteria. The results found that aqueous extracts inhibited harmful microbes [134].

A positive effect of pumpkin has been observed by investigating its antimicrobial activity against *Staphylococcus aureus*, *Bacillus subtillus*, *Escherichia coli,* and *Pseudomonas*. *aeruginosa*. Three different solvents were used to prepare the extracts: water, chloroform, and alcohol. The results showed that the alcohol extract was more powerful than both water and chloroform extracts. *Staphylococcus aureus* was sensitive to all extracts. Recently, the novel antimicrobial activity of ultrasonicated spinach leaf extracts using random amplification of polymorphic DNA (RAPD) markers and electron microscopy against both Gram-positive and Gram-negative bacteria has been revealed [134]. RAPD is an emerging technique used for diagnostic mutation detection within a genome. The range of the minimum inhibitory concentrations (MICs) of the extracted leaf spinach antimicrobial substances against *Escherichia coli* and *Staphylococcus* *aureus* was observed between 60 and 100 mg/mL. The optimal extraction conditions were at 45 °C, ultrasound power of 44%, and an extraction time of 23 minutes. The study showed that the treated bacterial cells appeared to be damaged by a reduction in cell number. In fact, it was inferred that spinach leaf extracts exert bactericidal activity by inducing mutations in DNA and causing cell wall disruptions.

## 7. Conclusions

In summary, plant extracts showed strong antioxidant capacity both in vitro and in vivo, and the extracts can be considered a good source of natural antioxidants and antimicrobials. Polyphenol extraction from plants using fast and appropriate techniques is a low-cost method due to the reduction in the amount of solvent used, in addition to avoiding the need for longer extraction times compared to the conventional extraction method. Moreover, natural bioactive compounds have been found to interfere with and prevent all kinds of cancer. Flavonoids have been shown to work as anti-tumor (benign, melanoma) agents involving a free radicals quenching mechanism (i.e., OH, ROO). In fact, many studies have shown that flavonoids play significant multiple roles including mutagenic, cell damage, and carcinogenic, due to their acceleration of different aging factors. In addition to antioxidant activity, the inhibition of cancer development by phenolic compounds relies on a number of basic cellular mechanisms. More comprehensive studies related to these compounds will enhance pharmaceutical exploration in the field of carcinogenic disease prevention.

## Figures and Tables

**Table 1:** Comparison of methods for assessing antioxidant capacity based upon mechanism, endpoint, quantitation method, and whether the assay is adaptable to measure lipophilic and hydrophilic antioxidants.

Antioxidant Assay	Mechanism	Endpoint	Quantification	Lipophilic and Hydrophilic AOC
ORAC	HAT	Fixed time	AUC	Yes
TRAP	HAT	Lag phase	IC50 lag time	No
FRAP	SET	Time varies	∆OD fixed time	No
TEAC	SET	Time varies	∆OD fixed time	Yes
DPPH	SET	IC50	∆OD fixed time	No
LDL oxidation	SET	Lag phase	Lag time	No

## Supplementary Materials

The following are available online at www.mdpi.com/2223-7747/6/4/42/s1.
